# First outbreak of Zika virus in the continental United States: a modelling analysis

**DOI:** 10.2807/1560-7917.ES.2017.22.37.30612

**Published:** 2017-09-14

**Authors:** Giovanni Marini, Giorgio Guzzetta, Roberto Rosà, Stefano Merler

**Affiliations:** 1 Department of Biodiversity and Molecular Ecology, Research and Innovation Centre, Fondazione Edmund Mach, San Michele all’Adige (Trento), Italy; 2 Fondazione Bruno Kessler, Trento, Italy

**Keywords:** ZIKV, outbreak, vector borne diseases, mathematical modelling

## Abstract

Since 2015, Zika virus (ZIKV) has spread throughout Latin and Central America. This emerging infectious disease has been causing considerable public health concern because of severe neurological complications, especially in newborns after congenital infections. In July 2016, the first outbreak in the continental United States was identified in the Wynwood neighbourhood of Miami-Dade County, Florida. In this work, we investigated transmission dynamics using a mathematical model calibrated to observed data on mosquito abundance and symptomatic human infections. We found that, although ZIKV transmission was detected in July 2016, the first importation may have occurred between March and mid-April. The estimated highest value for R_0_ was 2.73 (95% confidence interval (CI): 1.65–4.17); the attack rate was 14% (95% CI: 5.6–27.4%), with 15 (95% CI: 6–29) pregnant women involved and a 12% probability of infected blood donations. Vector control avoided 60% of potential infections. According to our results, it is likely that further ZIKV outbreaks identified in other areas of Miami-Dade County were seeded by commuters to Wynwood rather than by additional importation from international travellers. Our study can help prepare future outbreak-related interventions in European areas where competent mosquitoes for ZIKV transmission are already established.

## Introduction

Zika virus (ZIKV) has recently emerged as a significant threat to public health worldwide. Transmission of ZIKV to humans is thought to occur mainly via bites of *Aedes aegypti* mosquitoes; however, other mosquito species, such as *Ae. albopictus*, have been demonstrated as potential vectors [1,2], and other routes of infection are possible, including sexual transmission [3], blood transfusion [4] and vertical transmission in both humans [5] and mosquitoes [6]. Although infection is usually asymptomatic or mild [7], a causal link with congenital birth defects has been established, and a strong association exists with Guillain-Barré syndrome [8], a severe neurological condition. The low frequency of severe complications has been counterbalanced by a rapid geographic spread of the virus, which led the World Health Organization to declare a public health emergency of international concern (PHEIC) from 1 February to 18 November 2016. ZIKV was originally identified in Africa in 1947 [9], but the first large outbreak was not reported before 2007 in Micronesia [9], followed by further outbreaks in 2013 and 2014 in French Polynesia [10] and other Pacific Islands [7]. After the first cases were notified in Brazil in March 2015, ZIKV spread throughout South and Central America, with the notable exception of Uruguay and continental Chile, within 18 months [11,43].

In Europe, locally transmitted cases of ZIKV have not occurred to date. *Ae. aegypti* is currently present only on Madeira (Portugal) and around the Black Sea [12]; the much more widespread *Ae. albopictus* has a similar competence for ZIKV at 27°C, but none at 18 °C [2]. For these reasons, the risk of local transmission in temperate climate regions has been estimated to be minor [13,14], but the possibility of outbreaks transmitted by *Ae. albopictus* cannot be ruled out in warmer areas, allowing both greater vector densities and higher competence. Indeed, 11% of the European population is estimated to live in areas suitable for a ZIKV epidemic, mostly concentrated in the Mediterranean basin [14].

In the continental United States (US), the first outbreak of ZIKV was recorded at the end of July 2016 in the Wynwood neighbourhood of Miami-Dade County, Florida [15]. In mid-August, mid-September and mid-October, three additional outbreaks in other areas of the same county (South Miami Beach, North Miami Beach and Little River) were identified. On 9 December 2016, the Florida Department of Health declared to have cleared active ZIKV transmission from all identified areas, which counted ca 250 locally acquired infections overall [16], and the area has been Zika-free since then.

In this work, we analysed the transmission dynamics of the Wynwood outbreak using a mathematical model calibrated to outbreak data, and we assessed the efficacy of the implemented vector control measures in containing viral transmission. Results from this analysis provide useful insights for prevention and control of possible future outbreaks in European areas.

## Methods

The outbreak under study involved an area of approximately 2.6 km^2^ [15], with a population of 7,725 inhabitants [17]. Health authorities identified 21 locally transmitted symptomatic cases, with onset of illness ranging from 26 June to 5 August 2016.

We modelled the mosquito abundance over time *M(t)* by considering a fixed mortality rate μ and a time-dependent recruitment rate ψ(*t*) (defined as the average number of adult female mosquitoes produced by a single female adult) previously estimated on *Ae. aegypti* populations from Miami [18]. A density D of ca 1,560 female mosquitoes per hectare was estimated on $\overset{-}{t}$ = 26 July, before vector control treatments [15], in the following equation, using parameter values reported in Table 1: D *= K/aπr^2^*.

**Table 1:** Model parameters for Zika virus outbreak, Wynwood, 2016

Parameter	Interpretation	Value	Source
*K*	Female mosquitoes per trap per day before interventions	30	[15]
*a*	Capture rate (%/day)	2.46	[40]
*r*	Flight range for *Aedes aegypti* (in m)	50	[41]
*ρ*	Reduction in mosquito abundance following treatments (%)	75	[15]
*p_MH_*	Probability of transmission from mosquito to human per bite	0.214	[1]
*p_HM_*	Probability of transmission from human to mosquito per bite	0.767	[1]
*θ_M_*	Mosquito incubation period (days)	Gamma distribution (μ = 10.5, σ = 0.5)	[19]
*L_H_*	Latency before symptom development (days)	Gamma distribution (μ = 5.9, σ = 0.5)	[19]
*α_H_*	Symptomatic period (days)	Normal distribution (μ = 4.5, σ = 0.75)	[19]
*t_i_*	Duration of asymptomatic infection (days)	Uniform (range 7–13)	[42]
*q*	Probability of being asymptomatic	0.8	[4,7]
*μ_M_*	*Ae. aegypti* death rate (1/days)	0.1	[18]
*ψ(t)*	*Ae. aegypti* recruitment rate (1/days)	μ_M_ (1+0.25 cos(2π/365 (t-90.89))	[18]
*d*	Reporting probability for symptomatic individuals	0.1	[19]
*T_0_*	Date of index case importation	18 March (95% CI: 1 March–15 April)	Calibrated
*b*	Mosquito biting rate (1/days)	0.058 (95% CI: 0.055–0.061)	Calibrated

CI: confidence interval.

We modelled the mosquito abundance over time *M(t)* by considering a fixed mortality rate μ_M_, a time-dependent recruitment rate ψ(*t*) previously estimated on *Ae. aegypti* populations from Miami [18] and their density *D*. Specifically, we applied the following system to compute the mosquito abundance on each day *t*:

$M{\left(t\right)}^{'}=\psi \left(t\right)M\left(t\right)-\mu_{M}M\left(t\right)$

$M\left(\overset{-}{t}\right)=D$

**Figure 1:**
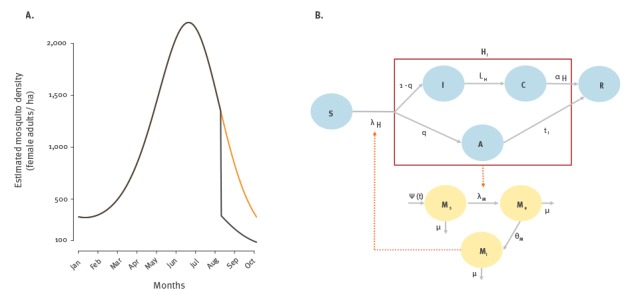
Modelled mosquito abundance and transmission model for Zika virus outbreak, Wynwood, 2016

We modelled the interventions by imposing a sudden decrease of the mosquito population size by ρ = 75% after 6 August, as suggested by the entomological data presented in [15] (Figure 1, panel A).

A. Predicted mosquito abundance over time in the study area. Black: 75% reduction in mosquito abundance following 6 August due to vector control interventions; orange: no vector control implemented.

B. Model flow chart for ZIKV transmission in humans (blue) and mosquitoes (yellow). Compartments: A: permanently asymptomatic humans; I: asymptomatic humans who will develop symptoms; C: symptomatic humans; M_e_: exposed mosquitoes; M_i_: infectious mosquitoes; M_s_: susceptible mosquitoes; R: humans who recovered after symptoms; S: susceptible humans. Parameters: λ_H_ and λ_M_ are the force of infection for humans and mosquitoes respectively and are computed as λ_H_ = b p_MH_ M_i_/H and λ_M_ = b p_HM_ H_i_/H, with H being the total human population in Wynwood; Ψ(t) is the mosquito recruitment rate, given by Ψ(t) = ψ(t) (M_s_ + M_e_ + M_i_). See Table 1 for parameter values and references.

Transmission dynamics of ZIKV in humans and mosquitoes were modelled according to the compartmental scheme reported in Figure 1, panel B. Susceptible humans (*S*) contract ZIKV from bites of infectious mosquitoes. A fraction *q* of infected individuals will remain asymptomatic (*A*) for the entire duration of viraemic infection *t_i_*, after which they will recover (*R*); the remaining fraction (1 − *q*) will remain temporarily asymptomatic (*I*) before developing symptoms (*C*) and will then recover (*R*). Recovered individuals are no longer infectious and become immune to reinfections. We assumed that infected humans (*H_i_*) are infectious regardless of apparent clinical symptoms. Symptomatic individuals have an overall probability *d* of being detected at some point. Susceptible mosquitoes (*M_s_*) can become infected (*M_e_*) after biting infectious humans; in such cases, they will become infectious to humans (*M_i_*) after an extrinsic incubation period and for the rest of their life. Given the very small rates of vertical transmission [6], we assumed that all mosquitoes newly emerged from their breeding site are susceptible.

To take into account the uncertainty surrounding the epidemiological parameters, we ran a stochastic version of the model proposed in Figure 1, panel B. We implemented an agent-based model representing the 7,725 inhabitants of the Wynwood neighbourhood, following an approach similar to the one presented in [19]. On each day, susceptible humans move to the infectious compartments based on a Poisson sample with a rate equal to the force of infection λ_H_; infected humans are subject to a binomial probability *q* of remaining asymptomatic. Asymptomatic individuals will remain infectious for a time *t_i_* sampled from a uniform distribution with a range of 7–13 days; symptomatic individuals will show their symptoms after a time L_H_ drawn from a gamma distribution with a mean of 5.9 days and shape parameter 0.5. Symptoms will last until recovery for a time α_H_, sampled from a normal distribution with a mean of 4.5 days and standard deviation 0.75. Vector dynamics were implemented as a stochastic stage-structured model representing the three possible epidemiological stages of the vector: susceptible, exposed and infected. See Table 1 for details on model parameters and the corresponding references for the adopted probability distributions.

We assumed that the Wynwood outbreak was initiated by a single index case imported in the area via international travel at time *T_0_*. Two free model parameters (*T_0_* and the mosquito biting rate *b)* were estimated by fitting the model-predicted weekly number of reported symptomatic cases to the observed cases. We used a Markov chain Monte Carlo (MCMC) approach with a standard Metropolis–Hastings algorithm with 100,000 iterations, uniform priors (range for T_0_: 1 January–20 June; range for b: 0–1) and a Poisson likelihood. We used the parameter values accepted by the algorithm as the posterior distributions, which were used to compute model predictions.

We computed the initial reproduction number R_0_(t) from the following formula [20]:

$R_{0}(t)=\frac{b^{2}\cdot p_{HM}\cdot p_{MH}}{\mu_{M}\cdot t_{i}}\cdot \frac{M(t)}{H}\cdot \frac{\theta_{M}}{\theta_{M}+\mu_{M}}$,

using the posterior distribution of the mosquito biting rate *b*, the modelled mosquito abundance *M(t)* and the epidemiological parameter values for ZIKV (Table 1). $R_{0}(t)$ represents a measure of the invasion potential of an infectious host introduced in a fully susceptible host population on day *t*. We also computed the effective reproduction number *R_e_*(t) as the product of *R_0_*(t) and the fraction of available susceptible individuals in the population [21]. *R_e_*(t)represents the residual transmission potential of an epidemic as the reservoir of susceptible individuals is depleted by the process of infection and recovery.

We also estimated the number of infected pregnant women and blood donors based on the model-predicted prevalence of human infections over time. Data on fertility [17] and blood donation [22] rates by age and ethnicity were adapted to the demographic structure of Miami-Dade County [17]. We considered all blood donations occurring between T_0_ and 27 July, when health authorities suspended blood collection from the area [23].

We evaluated the robustness of the proposed model (‘baseline model’) by fitting alternative model structures after varying some of the key assumptions: (i) We considered a broader mosquito flight range *r* of 82.5 m [24] in the estimation of the mosquito abundance (model M1); (ii) we assumed a relative reduction in mosquito abundance due to the vector control treatments (ρ) as a free model parameter sampled with a uniform prior with a range of 0–100% (model M2); (iii) we let the mosquito biting rate *b* vary over time, according to the same temporal dependence assumed for the recruitment rate [18] (model M3); (iv) we evaluated a model where the reporting rate *d* was a free model parameter with a uniform prior of 0–100% (model M4). Models were ranked according to the deviance information criterion (DIC) [25].

## Results

**Figure 2:**
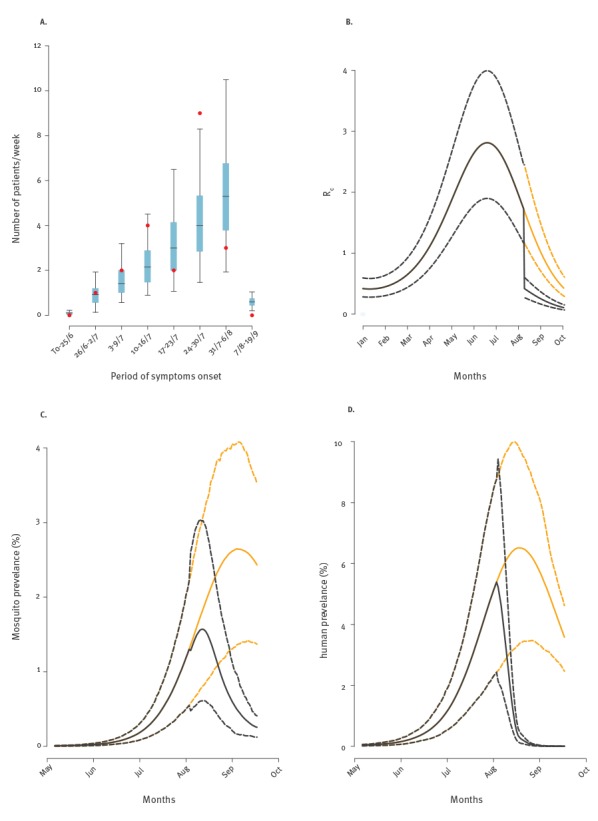
Reported cases, R_0_, and viral prevalence in humans as predicted by the model for Zika virus outbreak, Wynwood, 2016

The baseline model was able to reproduce the number of cases by date of symptom onset (Figure 2, panel A), as reported by health authorities [15]. The arrival of the index case was estimated between early March and mid-April (average: 18 March; 95% confidence interval (CI): 1 March–15 April), approximately corresponding to the dates when R_0_(t) approached the epidemic threshold (average: 28 March; 95% CI: 8 March–22 April). We estimated R_0_ to vary from a minimum of 0.4 (95% CI: 0.24–0.61) during winter to a maximum of 2.73 (95% CI: 1.65–4.17) in mid-June (Figure 2, panel B).

A. Predicted weekly number of reported symptomatic cases by date of symptom onset. Red dots represent observations [15] whereas boxplots indicate quantiles of the corresponding model-predicted distribution at 2.5%, 25%, 50% 75% and 97.5%.

B. Model estimates of the initial reproductive number for ZIKV in Wynwood over time.

C. Model-predicted prevalence of ZIKV in mosquitoes.

D. Model-predicted prevalence of ZIKV in human hosts.

Solid lines: mean values; dashed lines: 95% confidence interval; black: 75% reduction in mosquito abundance following 6 August due to vector control interventions; orange: no vector control implemented.

The total estimated number of human infections was 1,112 (95% CI: 436–2,120), corresponding to an average attack rate of 14.4% (95% CI: 5.6–27.4%). We estimated 15 (95% CI: 6–29) pregnant women to have been infected during the outbreak and a probability of 12% that at least one infectious individual donated blood before 27 July. The low occurrence of viraemic blood donors is due to smaller donation rates among the Hispanic population [22], the main ethnicity in Miami-Dade [17], and to the low (< 4%) predicted ZIKV prevalence until July.

According to data from the American Community Survey [26], incoming commuters represent ca 44% of the Miami-Dade population; of this proportion, the large majority (87%) are within-county commuters, while 13% come from outside the county (mostly from the neighbouring Broward County). This would correspond to ca 5.7% of infections (13% of 44%), i.e. ca 1 in 18, in commuters from outside the county and indeed, one of the 21 symptomatic patients reported in the outbreak was a commuter from Broward County identified via workplace investigation [15]. About 453 infections occurred in commuters resident in other parts of Miami-Dade; for comparison, only 208 travel-related ZIKV cases were recorded in Miami-Dade as of 19 September [27].

Vector control strategies were crucial for containing virus circulation by rapidly abating the average value of R_e_ from 1.49 to 0.37, with a sharp reduction of infection prevalence in both vectors and humans (Figure 2, panels C and D). In the absence of interventions, the model suggested that R_e_ would have remained above the critical threshold until the end of August, resulting in 55 (95% CI: 29.5–81.7) reported symptomatic infections and a total attack rate of 33% (95% CI: 16.7–49.3). Thus, according to our model, the implemented interventions have prevented ca 60% of all potential cases. Table 2 shows a sensitivity analysis of the expected total number of reported symptomatic cases, attack rate, and fraction of prevented infections for alternative scenarios on the assumed reduction in mosquito abundance allowed by the interventions. We estimated that even a treatment with low effectiveness, corresponding to a reduction of 25% of the vector population, would significantly reduce the expected number of infections by almost 40%.

**Table 2:** Effectiveness of alternative scenarios of vector control interventions on Zika virus infections, Wynwood, 2016

Reduction of mosquito abundance (6 August)	Total number of reported symptomatic cases		Total attack rate		Fraction of prevented infections compared with no intervention
	n	95% CI	%	95%CI	%
0% (no intervention)	55	29.5–81.7	33.0	16.7–49.3	0
25%	33.7	17.1–57.8	21.4	10.9–37.4	35.1
50%	26.5	11.4–48.8	17.0	6.2–31.7	51.5
**75%**	**22.6**	**9.2–42.2**	**14.4**	**5.6–27.4**	**56.4**
90%	21.3	9.7–35.8	11.7	5.4–20.3	64.5
75%*^a^*	18.1	9.2–33.2	11.5	5.9–20.8	65.1

CI: confidence interval.

The assumed effectiveness of implemented measures is shown in bold.

^a^ Reduction of mosquito abundance occurring on the same day of outbreak detection (23 July).

Table 3 shows a summary of results obtained with alternative assumptions on the model structure and parameter values. While the baseline model had the best performance in terms of DIC score, the overall qualitative conclusions were robust with respect to these variations, with alternative models suggesting an even earlier introduction of the virus and a slightly higher attack rate. The assumptions of ρ = 75% and *d* = 10% for the baseline model M0 were compliant with the estimates computed for models M2 and M4, respectively, where these parameters were estimated through the MCMC procedure.

**Table 3:** Comparison between alternative models, Zika virus outbreak, Wynwood, 2016

Model	DIC	Average T_0_		Average b		Average attack rate		Average d		Average ρ	
		Date	95% CI	Days^− 1^	95% CI	%	95% CI	%	95% CI	%	95% CI
M0: baseline model	36.55	18 Mar	1 Mar–15 Apr	0.05	0.055–0.061	14.4	5.6–27.4	NA	NA	NA	NA
M1: model with increased flight range	36.84	20 Feb	23 Jan–24 Mar	0.093	0.088–0.100	17.4	4.1–36.6	NA	NA	NA	NA
M2: model with free vector control efficacy	36.92	16 Feb	21 Jan–21 Mar	0.058	0.054–0.061	15.3	4.2–28.0	NA	NA	85.4	60.1–99.9
M3: model with time-dependent biting rate	37.20	13 Feb	1 Feb–21 Mar	0.052	0.049–0.055	16.9	4.2–35.7	NA	NA	NA	NA
M4: model with free reporting rate	39.15	10 Mar	10 Feb–3 Apr	0.057	0.054–0.060	15.0	4.2–27.3	12.8	10.0–19.7	NA	NA

CI: confidence interval; DIC: deviance information criterion; NA: not applicable.

## Discussion

In this work, we estimated that ZIKV was introduced to Wynwood between 3 and 5 months before the recognition of the outbreak by health authorities at the end of July. This long delay is justified because the infection prevalence in humans was small until June and because ZIKV symptomaticity [28] and reporting rates were low [19]. Our conclusion is supported by molecular-clock analyses of 32 ZIKV isolates from Florida, reported by the collaborative project nextstrain [29,30], which suggest that importation occurred between the end of February and the end of March. Model simulations indicate a likely extinction of transmission during winter even in the absence of interventions; however, we propose that undetected infections have occurred even after authorities declared the end of the outbreak on 19 September [27], given the sustained presence of infected mosquitoes. This prediction is consistent with the identification of locally transmitted cases in Miami-Dade as late as 28 December, almost three weeks after all active outbreaks in the area had been declared over [31]. Finally, our results suggest that the other three outbreaks in neighbouring areas of Miami-Dade County were probably seeded from Wynwood commuters rather than initiated from travel-related cases; a phylogenetic analysis of ZIKV isolates [29] supports this finding and shows that at most two viral clades were circulating in the four Florida outbreaks in 2016.

Our estimate of the mosquito biting rate is in line with a recent study that found parity and blood meal frequency to increase with temperature [32]. In particular, 60% of female mosquitoes were parous at 24 °C and took blood meals at an average interval of 11.7 days, whereas a parity of 86% and an interval between blood meals of 9.8 days were found at 27 °C. These figures correspond to a rate between 0.051 and 0.088 bites per mosquito per day. We estimated an average biting rate of 0.058 per mosquito per day throughout the period March to September, when the average temperature in Miami is 26.1 °C [33]. Because data on the biting rate at lower temperatures was not available, we did not include its temperature dependence as a possible driver of temporal variations in the transmission risk; however, the model assuming a seasonal dependency in the biting rate resulted in a similar quality of fit, an earlier estimated date of importation and a slightly higher attack rate. Other model parameters may be influenced by seasonal variations in temperature, with possibly shorter incubation periods (as in the case of dengue [34]) and increased transmission rates [2] during warmer months. Data for natural history parameters of ZIKV are insufficient to factor the temperature dependence in our model. Nonetheless, by analogy to what we found for the time-dependent biting rate, we expect that a further concentration of transmission in summer months would increase the estimated attack rate and push further back the date of introduction.

We estimated a peak value for R_0_ of ca 2.7, well within the range of other estimates for outbreaks in the Pacific and the Americas [14,19,35]. Previously published predictions for European areas with endemic *Ae. albopictus* populations also suggest potential values for R_0_ below 3 in the large majority of sites [14]. Therefore, the Wynwood outbreak is a relevant case study for potential future ZIKV transmission in Europe and can provide useful insights for prevention and control.

In Wynwood, about two weeks elapsed between recognition of local transmission on 23 July and a massive reduction in captured mosquitoes on 6 August [15]. These timely measures were successful in containing the attack rate below 15%. Even if effective interventions were immediately deployed on the day when local transmission was detected, the attack rate would still be above 11%. Because ZIKV is transmitted in a population for a long time before detection, reactive control measures are insufficient to prevent a large number of infections. A more effective approach would require the application of preventive measures with the aim of keeping R_0_ below the epidemic threshold. A recent study conducted on European *Ae. albopictus* showed that integrated vector control strategies can halve the mosquito abundance (and therefore R_0_) compared with sites where no intervention is implemented [36]. This reduction would not be sufficient to completely eliminate the risk of local transmission in the most exposed areas, but it would greatly limit the time window over which an epidemic is possible and, most importantly, its potential size, thereby reducing the risk of seeding further outbreaks in neighbouring areas. Furthermore, preventive control of mosquito populations would simultaneously reduce the risks of other mosquito-borne infections such as chikungunya and dengue [37].

Our conclusions are subject to some unknowns on ZIKV epidemiology, such as the role of asymptomatic infections, sexual transmission and spatial dynamics, along with uncertainties in parameter values and possible drifts in vector competence following adaptations of the viral genome [38]. The implications of this study for control are expected to be robust with respect to these uncertainties, since they depend on the silent transmission of ZIKV in the early months after importation, which is now a well-established trait of this emerging infection [39].

## References

[1] Chouin-Carneiro T Vega-Rua A Vazeille M Yebakima A Girod R Goindin D Differential susceptibilities of Aedes aegypti and Aedes albopictus from the Americas to Zika virus. PLoS Negl Trop Dis. 2016;10(3):e0004543. 10.1371/journal.pntd.0004543 26938868 PMC4777396

[2] Heitmann A Jansen S Lühken R Leggewie M Badusche M Pluskota B Experimental transmission of Zika virus by mosquitoes from central Europe. Euro Surveill. 2017;22(2):30437. 10.2807/1560-7917.ES.2017.22.2.30437 28106528 PMC5404485

[3] Mansuy JM Dutertre M Mengelle C Fourcade C Marchou B Delobel P Zika virus: high infectious viral load in semen, a new sexually transmitted pathogen? Lancet Infect Dis. 2016;16(4):405. 10.1016/S1473-3099(16)00138-9 26949027

[4] Musso D Nhan T Robin E Roche C Bierlaire D Zisou K Potential for Zika virus transmission through blood transfusion demonstrated during an outbreak in French Polynesia, November 2013 to February 2014. Euro Surveill. 2014;19(14):20761. 10.2807/1560-7917.ES2014.19.14.20761 24739982

[5] Besnard M Lastere S Teissier A Cao-Lormeau V Musso D Evidence of perinatal transmission of Zika virus, French Polynesia, December 2013 and February 2014. Euro Surveill. 2014;19(13):20751. 10.2807/1560-7917.ES2014.19.13.20751 24721538

[6] Thangamani S Huang J Hart CE Guzman H Tesh RB Vertical transmission of Zika virus in Aedes aegypti mosquitoes. Am J Trop Med Hyg. 2016;95(5):1169-73. 10.4269/ajtmh.16-0448 27573623 PMC5094235

[7] Duffy MR Chen T-H Hancock WT Powers AM Kool JL Lanciotti RS Zika virus outbreak on Yap Island, Federated States of Micronesia. N Engl J Med. 2009;360(24):2536-43. 10.1056/NEJMoa0805715 19516034

[8] Krauer F Riesen M Reveiz L Oladapo OT Martínez-Vega R Porgo TV WHO Zika Causality Working Group Zika virus infection as a cause of congenital brain abnormalities and Guillain-Barré syndrome: systematic review. PLoS Med. 2017;14(1):e1002203. 10.1371/journal.pmed.1002203 28045901 PMC5207634

[9] Hayes EB Zika virus outside Africa. Emerg Infect Dis. 2009;15(9):1347-50. 10.3201/eid1509.090442 19788800 PMC2819875

[10] Oehler E Watrin L Larre P Leparc-Goffart I Lastere S Valour F Zika virus infection complicated by Guillain-Barre syndrome--case report, French Polynesia, December 2013. Euro Surveill. 2014;19(9):20720. 10.2807/1560-7917.ES2014.19.9.20720 24626205

[11] World Health Organization (WHO). New detection of mosquito-borne Zika virus infections, 2013 - 2016. Geneva: WHO. [Accessed: 13 Mar 2017]. Available from: http://www.who.int/emergencies/zika-virus/situation-report/zika-timeline-13-october-2016.png?ua=1

[12] Kraemer MU Sinka ME Duda KA Mylne AQ Shearer FM Barker CM The global distribution of the arbovirus vectors Aedes aegypti and Ae. albopictus. eLife. 2015;4:e08347. 10.7554/eLife.08347 26126267 PMC4493616

[13] Guzzetta G Poletti P Montarsi F Baldacchino F Capelli G Rizzoli A Assessing the potential risk of Zika virus epidemics in temperate areas with established Aedes albopictus populations. Euro Surveill. 2016;21(15):30199. 10.2807/1560-7917.ES.2016.21.15.30199 27104366

[14] Rocklöv J Quam MB Sudre B German M Kraemer MU Brady O Assessing seasonal risks for the introduction and mosquito-borne spread of Zika virus in Europe. EBioMedicine. 2016;9:250-6. 10.1016/j.ebiom.2016.06.009 27344225 PMC4972550

[15] Likos A Griffin I Bingham AM Stanek D Fischer M White S Local Mosquito-Borne Transmission of Zika Virus - Miami-Dade and Broward Counties, Florida, June-August 2016. MMWR Morb Mortal Wkly Rep. 2016;65(38):1032-8. 10.15585/mmwr.mm6538e1 27684886

[16] Florida Department of Health (DOH). Department of Health daily Zika update. Tallahassee: DOH; 9 Dec 2016. Available from: http://www.floridahealth.gov/newsroom/2016/12/120916-zika-update.html

[17] US Census 2010. Washington: United States Census Bureau. [Accessed: 13 Mar 2017]. Available from: http://www.census.gov/2010census/

[18] Robert MA Christofferson RC Silva NJB Vasquez C Mores CN Wearing HJ Modeling mosquito-borne disease spread in U.S. urbanized areas: the case of dengue in Miami. PLoS One. 2016;11(8):e0161365. 10.1371/journal.pone.0161365 27532496 PMC4988691

[19] Kucharski AJ Funk S Eggo RM Mallet H-P Edmunds WJ Nilles EJ Transmission dynamics of Zika virus in island populations: a modelling analysis of the 2013-14 French Polynesia outbreak. PLoS Negl Trop Dis. 2016;10(5):e0004726. 10.1371/journal.pntd.0004726 27186984 PMC4871342

[20] Manore CA Hickmann KS Xu S Wearing HJ Hyman JM Comparing dengue and chikungunya emergence and endemic transmission in A. aegypti and A. albopictus. J Theor Biol. 2014;356:174-91. 10.1016/j.jtbi.2014.04.033 24801860 PMC4109365

[21] Keeling M, Rohani P. Modeling infectious diseases in humans and animals. Princeton: Princeton University Press; 2008.

[22] Shaz BH James AB Hillyer KL Schreiber GB Hillyer CD Demographic patterns of blood donors and donations in a large metropolitan area. J Natl Med Assoc. 2011;103(4):351-7. 10.1016/S0027-9684(15)30316-3 21805814

[23] Florida Department of Health (DOH). Department of Health daily Zika update. Tallahassee: DOH; 29 Jul 2016. Available from: http://www.floridahealth.gov/newsroom/2016/07/072916-local-zika.html

[24] David MR Lourenço-de-Oliveira R Freitas RM Container productivity, daily survival rates and dispersal of Aedes aegypti mosquitoes in a high income dengue epidemic neighbourhood of Rio de Janeiro: presumed influence of differential urban structure on mosquito biology. Mem Inst Oswaldo Cruz. 2009;104(6):927-32. 10.1590/S0074-02762009000600019 19876569

[25] Spiegelhalter DJ Best NG Carlin BP van der Linde A Bayesian measures of model complexity and fit. J R Stat Soc B. 2002;64(4):583-639. 10.1111/1467-9868.00353

[26] County to county commuting flows for the United States and Puerto Rico: 2009-2013. Commuting flows. Table 1. Washington: United States Census Bureau; 20 Apr 2017. Available from: https://www2.census.gov/programs-surveys/commuting/tables/time-series/commuting-flows

[27] Florida Department of Health (DOH). Department of Health daily Zika update. Tallahassee: DOH; 19 Sept 2016. Available from: http://www.floridahealth.gov/newsroom/2016/09/091916-zika-update.html

[28] Lessler J Chaisson LH Kucirka LM Bi Q Grantz K Salje H Assessing the global threat from Zika virus. Science. 2016;353(6300):aaf8160. 10.1126/science.aaf8160 27417495 PMC5467639

[29] Nextstrain. Real-time tracking of Zika virus evolution. [Accessed 13 Mar 2017]. Available from: http://www.nextstrain.org/zika/

[30] Neher RA Bedford T nextflu: real-time tracking of seasonal influenza virus evolution in humans. Bioinformatics. 2015;31(21):3546-8. 10.1093/bioinformatics/btv381 26115986 PMC4612219

[31] Florida Department of Health (DOH). Department of Health daily Zika update. Tallahassee: DOH; 28 Dec 2016. Available from: http://www.floridahealth.gov/newsroom/2016/12/122816-zika-update.html

[32] Goindin D Delannay C Ramdini C Gustave J Fouque F Parity and longevity of Aedes aegypti according to temperatures in controlled conditions and consequences on dengue transmission risks. PLoS One. 2015;10(8):e0135489. 10.1371/journal.pone.0135489 26258684 PMC4530937

[33] National Weather Service Forecast Office. Miami-South Florida. Silver Spring: National Oceanic and Atmospheric Administration. [Accessed 13 Mar 2017]. Available from: https://www.weather.gov/climate/index.php?wfo=mfl

[34] Focks DA Haile DG Daniels E Mount GA Dynamic life table model for Aedes aegypti (diptera: Culicidae): simulation results and validation. J Med Entomol. 1993;30(6):1018-28. 10.1093/jmedent/30.6.1018 8271243

[35] Nishiura H Mizumoto K Villamil-Gómez WE Rodríguez-Morales AJ Preliminary estimation of the basic reproduction number of Zika virus infection during Colombia epidemic, 2015-2016. Travel Med Infect Dis. 2016;14(3):274-6. 10.1016/j.tmaid.2016.03.016 27060613

[36] Baldacchino F Bussola F Arnoldi D Marcantonio M Montarsi F Capelli G An integrated pest control strategy against the Asian tiger mosquito in northern Italy: a case study. Pest Manag Sci. 2017;73(1):87-93. 10.1002/ps.4417 27539880

[37] Guzzetta G Poletti P Montarsi F Baldacchino F Capelli G Rizzoli A Assessing the potential risk of Zika virus epidemics in temperate areas with established Aedes albopictus populations. Euro Surveill. 2016;21(15):30199. 10.2807/1560-7917.ES.2016.21.15.30199 27104366

[38] Pettersson JH Eldholm V Seligman SJ Lundkvist Å Falconar AK Gaunt MW How Did Zika Virus Emerge in the Pacific Islands and Latin America? MBio. 2016;7(5):e01239-16. 10.1128/mBio.01239-16 27729507 PMC5061869

[39] Faria NR Azevedo RDSDS Kraemer MUG Souza R Cunha MS Hill SC Zika virus in the Americas: Early epidemiological and genetic findings. Science. 2016;352(6283):345-9. 10.1126/science.aaf5036 27013429 PMC4918795

[40] Maciel-de-Freitas R Eiras AE Lourenço-de-Oliveira R Field evaluation of effectiveness of the BG-Sentinel, a new trap for capturing adult Aedes aegypti (Diptera: Culicidae). Mem Inst Oswaldo Cruz. 2006;101(3):321-5. 10.1590/S0074-02762006000300017 16862330

[41] Bergero PE Ruggerio CA Lombardo R Schweigmann NJ Solari HG Dispersal of Aedes aegypti: field study in temperate areas using a novel method. J Vector Borne Dis. 2013;50(3):163-70. 24220074

[42] Lessler J Ott CT Carcelen AC Konikoff JM Williamson J Bi Q Times to key events in Zika virus infection and implications for blood donation: a systematic review. Bull World Health Organ. 2016;94(11):841-9. 10.2471/BLT.16.174540 27821887 PMC5096355

[43] Zhang Q Sun K Chinazzi M Pastore Y Piontti A Dean NE Rojas DP Spread of Zika virus in the Americas. Proc Natl Acad Sci USA. 2017;114(22):E4334-43. 10.1073/pnas.1620161114 28442561 PMC5465916

